# Identifying Barriers and Place-Based Solutions to Physical Activity for Older Adults in East Tennessee

**DOI:** 10.13023/jah.0703.08

**Published:** 2025-09-01

**Authors:** Lydia Hoskins, Jodi Southerland

**Affiliations:** East Tennessee State University; East Tennessee State University

**Keywords:** Aged, Appalachia, physical activity, physical exercise, public health, social determinants of health

## Abstract

**Introduction:**

Older adults in East Tennessee (TN), the fastest growing demographic, face significant health challenges, with 44% reporting four or more chronic conditions. The state ranks 45th in physical inactivity among older adults, exacerbating chronic disease risks, fall-related injuries, and mental health issues. Urban-rural disparities in East TN further complicate efforts to promote active living, particularly for rural residents.

**Purpose:**

This qualitative study explored socioecological barriers and solutions to increase physical activity (PA).

**Methods:**

In April 2024, two focus group were conducted in East TN, with a total of 11 participants composed of six community partners and five older adults. A semi-structured interview guide explored PA attitudes, beliefs, and behaviors and gathered recommendations for increasing physical activities among older adults. Data were analyzed thematically using a socioecological framework at individual, organizational, and community levels.

**Results:**

Barriers at the individual level included poor physical, psychological, and social health, and low digital literacy skills. Organizational-level barriers included limited capacity to support PA programming (e.g., staffing, facilities, and funding limitations) and overlapping community program efforts. Community level barriers included limited social support networks and inadequately built environmental features for active living. Solutions highlighted the role of relationships (e.g., peer-to-peer, trusted facilitators) at the individual level, tailored programming and marketing efforts at the organizational level, and leveraging local resources and multi-system collaborations at the community level.

**Implications:**

The findings highlight socioecological factors contributing to physical inactivity in East TN older adults and identifies strategies to address them. These findings can inform sustainable, multi-systems interventions to promote PA in the region.

## INTRODUCTION

Physical activity (PA) is essential for lifelong physical and mental health, reducing the risk of chronic conditions and decreasing premature mortality.^1^,^2^ Yet, 28% of Americans >50 years and nearly one-third (30.9%) of individuals >65 years are physically inactive.^3^,^4^ Tennessee (TN) ranks 45th nationwide for physical inactivity among older adults (38.9%).^4^

By 2040, over 24% of East TN’s population is projected to be 65+, presenting unique aging challenges.^5^ Approximately 43% of Medicare-enrolled older adults report >4 chronic conditions, and 39% experience functional limitations that hinder PA.^5^ Rural settings often lack dedicated exercise facilities and PA programs, and limited public transportation further restricts access to existing PA opportunities. East TN experiences high levels of social isolation — 10th highest in the U.S. — which promotes sedentary habits.^4^ This study applied the Social Ecological Model (SEM) to explore how individual, organizational, and community-level factors influence PA among older adults in East TN. It identifies place-specific barriers and potential solutions that emphasize relationships, tailored programming, and collaboration to promote active aging.

## METHODS

### Participants and Procedures

The East TN State University Institutional Review Board approved study procedures (0324.15e). Older adults (≥55 years) and community partners were screened by phone or email for eligibility by L.H; all interested individuals met the eligibility criteria. The older adult group included residents of the 33-county region served by University of Tennessee Extension. Residency was self-reported, and PA levels were assessed using adapted questions from the Behavioral Risk Factor Surveillance System.

Eligible community partners were professionals engaged in older adult health education, with priority given to those working in East TN. One academic participant from outside the region was included due to extensive rural fall prevention experience. Two focus groups — one with older adults and another with community partners — were conducted via Zoom by L.H. Eleven individuals participated: five older adults and six community partners from aging services, government, and academia. Separate semi-structured interview guides were designed to capture participants’ perceptions about PA behaviors, identify barriers to PA, and explore opportunities for collaboration to support PA engagement. Separate focus groups encouraged open dialogue and shared experience. All participants provided informed consent prior to the focus groups.

### Analysis

Focus group recordings were transcribed verbatim and verified for accuracy. Transcripts were initially coded line-by-line for internal (e.g., attitudes, beliefs, and behaviors) and external (e.g., environmental, systemic) factors, along with potential solutions. A list of *a priori* codes was developed to identify key facilitators, challenges, and solutions. Both authors independently coded the transcripts, resolving discrepancies through discussion. Data were organized in Microsoft Excel according to the finalized list of codes and categorized by SEM level.^6^ Weekly meetings ensured consistency and consensus in interpretation.

## RESULTS

Community partners included four men and two women. Older adults (n=5) ranged in age from 69 to 78 years (M=74, SD=3.74); these five older adults were composed of three women and two men, all managing at least one chronic condition. Analysis yielded eight themes related to place-specific barriers and solutions, organized by SEM levels (Tables 1 and 2).

### Individual-level barriers

Older adults cited physical limitations, such as age-related conditions, that influenced activity choices and engagement. One participant noted, “The arthritis in my left knee causes my leg to swell, but when I walk especially outdoors, even on uneven ground … I’m aware of that so I try to get up and move more,” highlighting how symptoms influence behavior.

Psychological and social health factors included fear of losing independence, the desire to age in place, and the motivation to take charge of one’s health versus perceived social judgment in gyms. Some felt uncomfortable using gym equipment, preferring senior centers where they felt more comfortable. The instructor-participant relationship (i.e. trust) also shaped engagement patterns.

Group support emerged as another influential factor. One older adult shared she only engaged in activity when encouraged by others, emphasizing the importance of peer influence. Intergenerational connections created through group exercise were also seen as meaningful. Limited digital literacy posed a barrier, with some older adults struggling to navigate online resources.

### Organizational-level barriers

Challenges included limited staffing, outdated equipment, aging facilities, and limited funding. Rural staffing shortages restricted outreach, often limiting participation to those who independently seek PA programs. One practitioner remarked, “No matter how much effort [he] put into preparing, sometimes no one shows up,” acknowledging the difficulty of engaging less motivated audiences. In some cases, overlapping programs competed for resources. Caregiving responsibilities also interfered with regular attendance, adding to retention challenges.

### Community-level barriers

Rurality and infrastructure limitations were major concerns. Manual labor and farming are viewed as primary forms of PA among rural older adults, making structured exercise feel unnatural. One practitioner noted that homebound older adults often rely solely on family or home health aides for support, contributing to isolation. Financial and technological disparities also shaped access. One practitioner shared that his grandparents struggled to find affordable health coaching, while another older adult noted “good insurance” improved service accessibility, demonstrating the impact of financial resources affect PA participation.

Limited broadband and public transportation were also cited as significant barriers, hindering access to PA programs in rural communities. Limited internet access also restricts awareness of available opportunities despite organizations’ promotion efforts. Many older adults still rely on newspapers for updates, highlighting a persistent communication gap.

One older adult noted, “The [gym] is a beautiful facility, it is far out [and not] practical … it’s not far for me to drive, and yes, I do drive, and but it is far out,” echoing the challenges of rural geography. A community partner contrasted a previous role in a community-centered parks department with their current urban position offering more diverse senior programs, highlighting rural-urban disparities in PA access.

#### Potential Solutions

Potential solutions spanned all levels, organized into four themes (Table 2).

### Individual-level solutions

Peer relationships and trusted facilitators were strong motivators for PA engagement and retention. Practitioners emphasized that group exercise programs fostered meaningful connections, often serving as the only social interaction some participants had each week. Instructors who offered physical modifications to improve accessibility, connected with participants before and after class, and built rapport were especially effective in supporting ongoing participation and retention.

### Organizational-level solutions

Tailoring programming to the community’s preferences and offering motivational incentives (e.g., small prizes, recognition) to encourage attendance were noted solutions. Participants suggested expanding outreach through digital and print marketing and holding programs in familiar, non-traditional exercise facilities like churches, banks, and farmers’ markets.

### Community-level solutions

Collaboration among local organizations was strongly endorsed to leverage local resources and strengthen cross-sector partnerships. One practitioner emphasized, “Partnerships are crucial for extending our reach across counties. Collaborating with Extension agents, Health Department educators, and universities allow us to maximize our impact and is cost-effective.”

Community faith nurses were identified as particularly effective for engaging older adults through church congregations. A grant-funded initiative was described in which live classes were streamed to homebound older adults, including those in public housing, illustrating how collaboration can overcome logistical barriers. While successful, the program highlighted ongoing rural challenges, including the need for staff support and improvements in broadband infrastructure.

## IMPLICATIONS

This study underscores the importance of engaging stakeholders to better understand the complex, multi-level factors influencing PA among older adults. These perspectives are essential for shaping relevant, sustainable strategies that support active aging. Consistent stakeholder engagement improves program design, increases community buy-in, and strengthens long-term impact.

TN ranks 43rd in older adult health, with high rates of physical and mental distress contributing to lower leisure-time PA.^4^ Existing literature highlights the value of understanding rural older adults’ perspectives and preferences—especially as they relate to aging-in-place, a concern closely tied to maintaining independence in daily activities, with perceptions of personal health and the surrounding environment being key influences.^7^,^8^ Creating age-friendly communities requires including older adults’ voices to ensure initiatives reflect lived experiences and actual needs, enhancing the well-being of rural older adults.

Physical and psychological factors (e.g., fear of falling and limited social support) were key barriers. These findings align with Self-Determination Theory, which emphasizes relatedness, autonomy, and intrinsic motivation in maintaining behavior change.^9^

Organizational and infrastructure barriers — like fragmented referral systems, inadequate transportation, and limited broadband — limit access. TN’s low investment in these areas hinders organizations’ ability to promote PA opportunities effectively.^7^ Collaboration across agencies can reduce redundancy, improve reach, and expand access, particularly for homebound or socially isolated individuals; addressing these gaps requires coordinated efforts across local, regional, and state partners. Delivering programs in trusted familiar community settings and fostering strong instructor-participant rapport can also enhance retention and comfort.

Though the sample size was small, it included diverse voices across experiences and PA levels. Data saturation was achieved, and findings offer valuable insight. Future studies should expand participation for broader generalizability. While Zoom provided accessibility, technical issues affected engagement for some, emphasizing the need for flexible, inclusive engagement methods. Implications for practice include co-designing programs with older adults to ensure local relevance and feasibility. Place-based approaches, rooted in community values and delivered through trusted networks, can enhance participation and sustainability. Strengthening connections among Extension, public health, aging services, and faith-based partners can further support a comprehensive, community-level response.

Promoting PA among rural older adults requires integrated solutions that reflect the complexity of their lived experiences. Aging-in-place requires holistic, community-based strategies that bridge individual, organizational, and environmental factors that are grounded in local voices and supported through strong, multi-sector partnerships.

**SUMMARY BOX**

**What is already known about this topic?**

Rural older adults experience profound health disparities, marked by high prevalence of chronic conditions and physical inactivity. Addressing inactivity is particularly challenging due to the place-specific landscapes (i.e., rural versus urban) and the multi-level barriers that exist.

**What is added by this report?**

This study adds to the literature by applying a socioecological framework to identify both internal and external, older adult-identified barriers to PA in a rural context. It highlights place-specific solutions that emphasize the importance of relationships, tailored programming, and cross-sector community collaboration. Findings reinforce the value of direct engagement with older adults and local stakeholders in designing sustainable, contextually relevant PA interventions.

**What are the implications for future research?**

Policy makers, researchers, and aging service organizations should solicit insights from older adults and community partners in the development of physical activity programming to ensure sustainability and relevance.

## Figures and Tables

**Table 1:** Socioecological Barriers to PA – Summary Quotes

Individual Level	
*Theme*	
*Physical Health*	
**55+ Focus Group Response**	I feel like the slug of the group. I’ve been doing absolutely nothing. I generally get between 4 and 10,000 steps a day. I used to be extremely active, my change in my circumstances has taken me away from anything I really wanted to do like that. I used to swim, I used to do the Yoga classes at the [gym]. I do have arthritis… think I still have quite a bit of strength, and I turned 69 this year, so. But I’m trying to get back into walk[ing], and I would say that right now the one of the biggest things is I am socially motivated…I am staying socially active. But no, I do not have a lot of people that that go out and do stuff. I really have a limited friend group here, a limited friend group that that would be active. If I get my raggedy tail up and go on back to the [gym], I’d be a lot probably a lot better, but that we’ll see if that can happen this spring. Anyway, it hasn’t happened up to this point.
**Community Partner Focus Group Response**	I think for the senior center, the pandemic created a barrier because we had people who were off of walkers, were physically active, and were attending exercise classes 3 or 4 times a week. Then they went through the pandemic, they were isolated at home and [were sedentary]. When they come back, *if* they came back, because there’s still a big fear in coming back to a place where there’s lots of people, they were then either on a cane or walker. We have been working with specifically some of those people to bring them back to the fitness level they were at before the pandemic.
*Psychological Health*	
**55+ Focus Group Response**	We’ve got to help people change their perspective…they think we’re gonna get out there and do jumping jacks, or you know something that seniors cannot do, and they don’t realize the benefits of stretching and the exercises for balance and that sort of thing…They’ve got a misconception about physical exercise that it’s hard, and that they’re not going to be able to do it, and it’s going to hurt. And so I think part of our challenge is to change their perspective.
**Community Partner Focus Group Response**	Another thing I hear sometimes when I’m talking to older adults about physical activity. I’ve heard that from older adults, you know, “Oh, I don’t want to go get on a machine. They’re all gonna look at me.” And so that perception of, you know, “I’m older, and shouldn’t be in here,” I think sometimes overplays the importance of them being active and stay inactive.
*Social Health*	
**55+ Focus Group Response**	The group exercise is...you don’t have to kind of go by individual machines and that type of thing. I think it is a good social way to trying to get getting involved with other people and established friends kinda in the same age group, and some that are about one third our age; the young mothers that are leaving their babies there and working out, and we can kind of do more by having people of all ages there. So I think that’s a benefit as well.
**Community Partner Focus Group Response**	So many of these older adults grew up, when everybody had a farm; everybody was out working… And so their perception, physical activity was, they were out in their yards, their gardens around the farm, working all day. Go[ing]to an exercise was not something that they grew up doing… That was just their nature of life… They don’t perceive that as something important until a lot of times they have a fall or something, and they go through PT, and they say, “You need to be moving and exercising.”…When I go out to senior centers I try to talk with them and see what’s going on …and even the ones that are opposite, they’ll be like, “Well, I just didn’t think I need to come here till I did.”
*Technological Literacy Skills*	
**55+ Focus Group Response**	I know you are doing canning, diabetes, and balance classes at community centers or libraries because I’m online. If you’re online, you see this stuff.
**Community Partner Focus Group Response**	The barrier of lack of information…some individuals don’t get [it]. Because we live in this world where everything is social media, and we use that to spread the word about, especially recreation, but you know our seniors just don’t have that. And so we’ve got to be conscious here at the [center] to make sure we have things in print that we give them.
**Organizational Level**	
*Limited Capacity to Support PA Programming*	
**55+ Focus Group Response**	Yeah, the problem with some of those... I mean, there’s only one of [the instructors]. Okay, [she] can’t physically be everywhere... There are many people out there teaching exercise classes that you have to pay for Tai Chi, you have to pay for certain Yoga classes, and you know they’re very set times and you’ll find those classes, you know, places like this the Central senior center. But trying to get into other areas. I mean [the instructor] competes with other programs.
**Community Partner Focus Group Response**	An arm of the aging program at the [facility] was going into low income, senior housing and doing fitness programming…and we would do flyers…[and] make the access as easy as possible. I mean, I would go on site…and knock door to door, you know, at the beginning of the week, and say, “I’m going to be here on Friday”. And I think…I just got to the point where I’m like, you know, I’m putting in 8 hours of preparation in this a week, and then I go over there for an hour, and there’s no one that shows up, no response, and that makes it really challenging. And so I just got to the point where I’m like…I’m one person, so I can only spread myself so thin, and I think that may have been a barrier, for when we were going to these other places—I could only have a relationship that was so deep with certain amount of people.
*Overlapping Community Program Efforts*	
**55+ Focus Group Response**	I’m a member of the senior center, and they do have a lot of activities that go on at Senior Center. And I do participate in some of those; I tend to go towards the [other gym facility] a little bit more, [I] am trying to blend the both. It has been difficult because of the timing.
**Community Partner Focus Group Response**	I don’t know if you see it as much, but like I see when I’m talking to people what they’ll be at the [senior center A], but they may go to the [senior center B], and they may go to the [senior center C] and dependent on where they live. They may go, you know to 2 of them, or all 3, and I don’t know if you all see that in [that] County, because it’s so much more expanse, you know. But you know I don’t know what y’all see. Do you all see where they maybe bounce around from both senior centers or the senior center in the one, you know depending on what’s going on?
**Community Level**	
*Rurality*	
**55+ Focus Group Response**	People may only have one vehicle, and somebody is taking that to work. You know, they’re in such a situation that they’d have no vehicles, even if they’re single.
**Community Partner Focus Group Response**	I am aware that transportation just with my own personal experience with my grandparents that it can be a barrier, but I think a number of things it could be access to just affordable or available like health coaching.
*Limited-Built Environment to Support PA*	
**55+ Focus Group Response**	I think it’s more difficult for people who live in rural areas to get to facilities, because [public transportation] doesn’t run in rural areas… but it’s difficult for some people to get out because of their family situation, or the fact that they live in a rural area that doesn’t have any type of public transportation at all.
**Community Partner Focus Group Response**	In [this] County we don’t have multiple facilities and… we don’t have all that. We have our one small senior center…you know, but in [a neighboring] county there are. So I think that community culture is a little more, you know…[the neighboring county] has parks and walkways... So I think there’s some of that where it’s a little more accessible and more the cultures more there for older adults to be out and be active in some of our other areas that I cover.

**Table 2:** Potential Solutions to Physical Inactivity – Summary Quotes

Individual Level	
*Theme*	
*Role of Relationships*	
**55+ Focus Group Response**	I have a recommendation. I think if you bring the social aspect along with an activity, because I feel that a lot of people in our age are socially deprived…they need to connect with people their own age. And so to me, having activity where you go out to have breakfast, and then you go for a little walk afterwards, or something like that would be nice, you know, because then you get your camaraderie of being able to talk to all the different people, and you go out and get some fresh air, and you work off the eggs at your own pace.
**Community Partner Focus Group Response**	Having again, those local connections make it viable, because trust is a big thing. To be able to deliver to this audience is sometimes hard to reach…I mean, if you gotta overcome these barriers with older adults, you know, there’s a lot of times they’re not confident in being physically active, and they’re intimidated to try it. There’s a fear of embarrassment. There’s a fear of injury. You know. There’s the activity aging cycle. As we get older, you know, we’re less active, we’re less able. And that just perpetuates being sedentary. And you’re less able to do these things if we can get the cycle moving in the other direction, where they see a little success. And that’s why I think with stay strong. We’ve been successful because these older adults are with peers, and they can relate versus trying to go to a you know, a traditional gym can be very intimidating. And then, they start to build those social relationships, not just with our instructors, but with each other... That’s a huge thing...so we’ve noticed, you know, these folks show up early. They stay around late and they’re excited to be there. So that’s if you can get them there, then you can start to build their trust. And with that local agent in their community. It just goes a very long way to success.
**Organizational Level**	
*Intentional Programming and Marketing Efforts*	
**55+ Focus Group Response**	What’s the idea of a health fair to have a health fair with all the different you know, booths [set] up that everybody in the community comes to offer what their services is. And then that’s a way of getting the information out, and everybody likes to go to a health thing gets little trinkets, even if you get a damn Band-aid, it’s still fun. You got something for free, you know. But to do a thing like that to get the information out, especially at the beginning of the of the nice season, and doing a place I don’t know where, but, you know, having all these health type of people come in and show off their products.
**Community Partner Focus Group Response**	In a world dominated by social media, which we use to promote recreation, many seniors are not plugged into these platforms. At the center, we need to ensure we provide printed materials to keep them informed. This is an important concern we must address.
**Community Level**	
*Leveraging Local Resources*	
**55+ Focus Group Response**	Now, if you could get the meeting to be at the [walking trail] and go for walk by the river, you know, and then you’re sometimes in the shade and sometimes not, and it’s a lovely scenery, and the location is good. I mean, people want to bring their dog. They can bring their dog if they want to. So, I think that has a lot to do with it. If you can find to have these classes and things in locations that are nice to get there outside. And you’re getting the fresh air and everything else on top of it.
**Community Partner Focus Group Response**	I don’t know… you may can talk on this more than, but you know I’ve always suggested if the senior centers [are] not connected, because some of ours are connected to Parks and Rec…can we somehow partner with parks and Rec, even if they’re not to get, you know, to actually hit some of those [rural] areas if they have a facility? Like in, you know, [the] city, they have…a place that…we partner with places like that where they’re in those outlying areas.
*Multi-systems Collaborations*	
**55+ Focus Group Response**	I believe, for rural areas [there] is an opportunity to have somebody they know and trust, and that might be somebody in their local church that they can walk to. It may be a retired nurse could kind of help pull it together for them, and they could do things as a church, as you know, people that could walk to church and can walk to church and walk around as well. They could plan some hikes and things together. So I think the rural churches could be a good fit.
**Community Partner Focus Group Response**	For the most part getting everybody together and just tackling that kind of main issue which is inactivity, lack of education on eating and lack of education, on moving and exercise…Connecting like-minded organizations, that kind of has a similar mission and bringing them together so that they can more effectively achieve their goal…purpose, or their mission… getting people to realize that, Hey, I know that you’re a nurse, or you know you’re at a senior living facility. Or you’re the director of a senior living facility… How can we collaborate? How can we come together to achieve this same goal? Collaborating with…key individuals that have influence, getting them in the same room [that are] trying to achieve the same mission, it [can] make me more effective in my job.
